# The Use of Colistin in Food-Producing Animals in Estonia—Vaccination as an Effective Alternative to Consumption of Critically Important Antimicrobials in Pigs

**DOI:** 10.3390/antibiotics10050499

**Published:** 2021-04-28

**Authors:** Marju Sammul, Kerli Mõtus, Piret Kalmus

**Affiliations:** 1 Institute of Veterinary Medicine and Animal Sciences, Estonian University of Life Sciences, Kreutzwaldi 62, 51006 Tartu, Estonia; kerli.motus@emu.ee (K.M.); piret.kalmus@emu.ee (P.K.); 2 State Agency of Medicines, Nooruse 1, 50411 Tartu, Estonia

**Keywords:** colistin, *Escherichia coli* vaccination, national statistics, pigs

## Abstract

Reducing the use of critically important antimicrobials in veterinary medicine is increasingly important to avoid the development and spread of antimicrobial resistance. The aim of this study was to analyse ten-year trends of colistin consumption in Estonia and to ascertain the possible association between *Escherichia (E.) coli* vaccination and colistin consumption in pig production. Colistin sales data (2010–2019) were collected from the wholesalers, allowing differentiation of target species. In Estonia, the amount of sold colistin increased constantly and almost doubled from 2010 to 2013, and decreased from 2013 to 2019 by 92.5% in total. On average across a ten-year study period, 89.7% of colistin was used in pig production. The number of sold doses of *E. coli* vaccines for pigs was very low before 2014 (<2000) and increased drastically to 2019 (362,000). According to linear time-series model with autoregressive integrated moving average (ARIMA) the consumption of colistin in pigs decreased on average by 0.23 mg/PCU for every 10,000 *E. coli* vaccine doses (95% CI −0.39, −0.06; *p* = 0.006) over ten years. This study revealed that in pig production, vaccination against *E. coli* strains contributes to the expected downward trend in colistin consumption.

## 1. Introduction

Lists of critically important antimicrobials (CIAs) according to their importance in human and veterinary medicine are created at the international, regional, and national level [1], and polymyxins are predominantly classified as the highest priority CIAs to human medicine [2,3]. One of these in clinical use is colistin (polymyxin E), which was discovered in 1949 [4] and has been used in both human and veterinary medicine [5,6]. In animals, colistin has been widely used in pig production [7,8] for the treatment and prevention of post-weaning diarrhea [9,10,11] caused by *E. coli. E. coli*-induced diseases are responsible for economic losses in pig production as they cause high morbidity and mortality among piglets [12]. In human medicine, due to the shortly afterwards discovered systemic toxicity, the use of colistin was limited [13] and the risk for the zoonotic transfer of resistance genes and resistant bacteria was estimated low. Therefore, colistin has been used extensively in veterinary medicine as group treatment or for the prevention of diseases [10] for many years.

The development of colistin resistance has been associated with the non-transferable genome-mediated mutation. In 2015, a plasmid-mediated gene, *mcr-1* was identified in gram-negative bacteria, such as *E. coli* and *Salmonella* spp., which made a transfer of polymyxin resistance easier. The *mcr-1* was found in several countries from various origins including farm animals, raw meat, and humans [14,15,16]. In Estonia, *mcr-1* was isolated from a pig slurry sample in 2015 [17]. In recent years, systemic colistin treatment in humans has been re-introduced. In 2016, the European Medicines Agency (EMA) updated the advice on the impact of colistin use for human and animal health and raised the level of the overall probability of transfer of colistin-resistant bacteria from low to high [18] and the World Health Organization (WHO) re-classified polymyxins as the highest priority CIAs for human medicine. Colistin has become an important antimicrobial for the treatment of infections caused by multidrug-resistant gram-negative bacteria [19,20]—the last-resort antimicrobial critically important for human health, which uses in animals should be restricted [3].

The global spread of colistin-resistant bacteria requires decisive action in reducing the use of colistin in livestock and particularly in pig production. Some countries like the Netherlands, which had significantly reduced their antimicrobial use (AMU) in animal production, had shown lowered *E. coli* resistance levels in pig production [21]. The major policy changes like the withdrawal of colistin as a feed additive are important steps on reducing colistin resistance and have shown great impact on the use of colistin in China after the ban of colistin use as a growth promoter in 2017 [22]. The European Union (EU) banned the use of all antimicrobial growth-promoters in animal feed for all EU countries already in 2006 [23]. Nevertheless, the total prohibition of some antimicrobials may not lead to the desired result, the demanding continuous reduction of CIAs may cause an increase in the use of other antimicrobials [24,25].

The increased antimicrobial resistance (AMR) among animal and human pathogens has promoted the search for alternatives. One of such is the herd-level application of vaccination programmes. Several studies have demonstrated the potential of vaccines to achieve a significant reduction of antimicrobial consumption in swine herds [26,27]. Vaccination of pigs against different pathogens as *Actinobacillus pleuropneumonia* [28], *Lawsonia intracellularis* [29], porcine circovirus type 2 [30,31], porcine reproductive and respiratory syndrome virus [32] can lead to the remarkable decrease of AMU. The effect of vaccination in swine herds on the use of polymyxins has been studied in combination with other alternatives [33]. However, we are not aware of any published studies addressing the association between *E. coli* vaccination of swine herds and the consumption of colistin.

The use of colistin and other CIAs is not prohibited in animals in Estonia. However, the instructions for the prudent use of antimicrobials are publicly available since 2012 [34] and the Estonian Ministry of Rural Affairs published the treatment guidelines with strong recommendations to avoid CIAs for the treatment of food-producing animals [35]. The comprehensive analysis revealing the time-trends of colistin consumption in Estonian production animals is missing. The aim of the study was to analyse the use of colistin in food-producing animals in the years 2010–2019 in Estonia and to identify the possible association between vaccination of piglets against *E. coli* and consumption of colistin in pigs.

## 2. Results

Out of polymyxins, only colistin sulfate was sold in Estonia during our observation period. In 2010–2019, five different nationally authorized colistin-containing veterinary medicinal products, indicated for oral administration to pigs, cattle (calves), and poultry, were sold. Three of them were sold throughout the 10-years study period. For two of them, there was a one-time sale in 2015 and in 2019.

In 2010–2019, vaccines for pigs from ATC group immunologicals for Suidae (QI09), containing *E. coli* component, were sold with two ATCvet codes: inactivated bacterial vaccines containing *E. coli* components with ATCvet code QI09AB02, and inactivated bacterial vaccines containing both *E. coli* components and *Clostridium* components with ATCvet code QI09AB08.

### 2.1. The Sales of Colistin by Animal Species in 2010–2019

The total sales of colistin both in kilograms and in mg/PCU increased from 2010 to 2013 and have decreased steadily since 2014 (Table 1). The proportion of colistin from all sold antimicrobials (% of total AM) was highest in 2013 (8.1%) and during 2015–2019 has ranged between 0.7–1.7%. The overall use of colistin decreased from 378 kg to 53 kg (by 86.0%) during the whole period.

**Figure 1:**
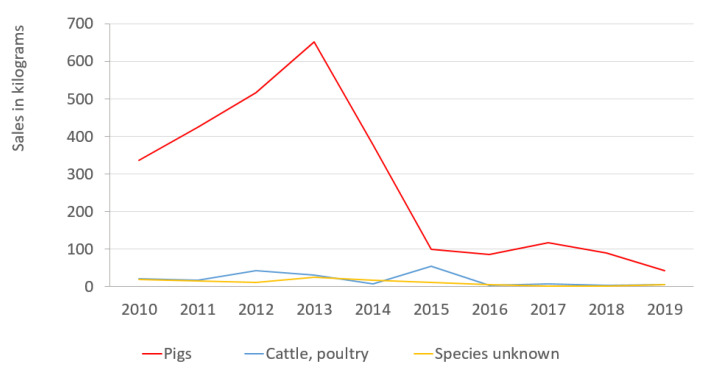
Sales of colistin (kg) by animal species in years 2010–2019, in Estonia.

In 2010–2019, the sales of colistin for pigs accounted for 60–93% of the total consumption of colistin, while on average 6.5% were sold for cattle and poultry (Table 1). The quantities of colistin used in pigs, almost doubled by 2013 compared to 2010 and thereafter, a sharp drop in 2014–2015. The colistin sales for pigs decreased from 2010 to 2019 by 87.5% in total (Figure 1). The sales for cattle and poultry fluctuated slightly, showing a decrease of 72.7% in total. The target species remained unknown for 3.8% of sold colistin across the ten-year period. The colistin sales for unknown species decreased by 75.0% in total (Table 1).

### 2.2. Association between Vaccination of Piglets Against E. coli and Consumption of Colistin in Pigs

**Figure 2:**
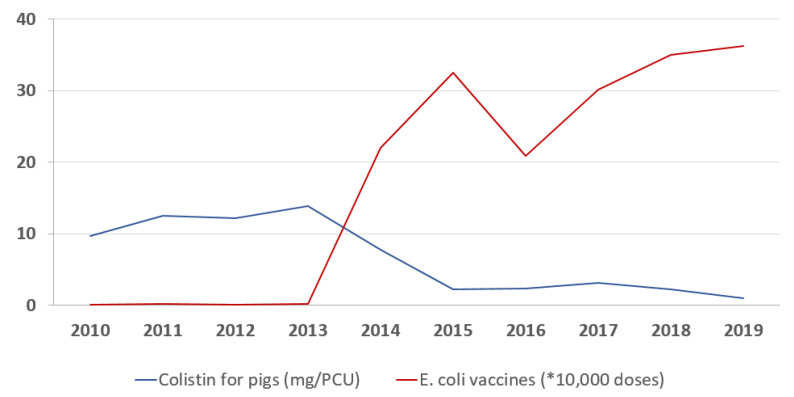
Consumption of colistin and *Escherichia coli* vaccines for pigs in 2010–2019 (* vaccines are presented in 10,000 doses, colistin is presented in mg/PCU).

In 2010–2013, the average number of piglets born in Estonia per year was 765,000 [36], while the number of sold doses of *E. coli* vaccines for pigs with ATCvet code QI09AB02 remained under 2000. From 2013 to 2019, the number of sold vaccine doses increased to 362,000 (Table 2, Figure 2). In 2013, the consumption of colistin in pigs was 13.88 mg/PCU (Table 2). In the first years of active vaccination, in 2014–2015, colistin use in pigs decreased to 7.77 mg/PCU and 2.20 mg/PCU respectively and remained considerably lower in 2015–2019 compared to 2010–2014 (Table 2, Figure 2).

According to the time-series ARIMA model the consumption of colistin decreased on average by 0.23 mg/PCU for every 10,000 vaccine doses (95% CI −0.39, −0.06; *p* = 0.006).

## 3. Discussion

Globally, major policy changes have followed the discovery of *mcr-1* and the reclassification of polymyxins as the highest priority CIAs for human medicine. The important step implemented in several countries worldwide is a withdrawal of colistin as a feed additive in animals. This has already resulted in a reduction in colistin use in China [22]. In Europe, between 2011 and 2018, sales of polymyxins decreased by 69.8% in European Union/European Economic Area (EU/EEA) countries overall [37]. There is little information available about how this decrease was achieved in various countries. Denmark, for example, uses for pigs the VetStat “yellow card initiative” monitoring system [25,38,39], which warns against the overuse of antimicrobials. There was a large drop in the use of polymyxins in Spain and Italy, on which a brief summary by country is published by the European Surveillance of Veterinary Antimicrobial Consumption (ESVAC) [40]. Estonia reduced sales of polymyxins remarkably and achieved in 2018 twice-lower level as the median in 31 European countries.

The present study revealed that a remarkable increase of colistin use from 2010 to 2013 in Estonia and a sharp reduction thereafter, was largely the result of changes in colistin use in pigs. In addition, we ascertained a significant association between increased vaccination of piglets against *E. coli* and reduction of colistin use. In addition to improved rearing conditions and feeding, vaccination of piglets against *E. coli* strains has been shown as an effective alternative to control post-weaning diarrhea at the farm-level [41]. However, in most cases, successful disease prevention and an expected decrease in AMU is not achieved by a single alternative measure. Raasch et al. [33] assessed the effectiveness of alternative measures in pig production, such as improvement of biosecurity, vaccination, improved feeding, and health care, and found a significant reduction in colistin consumption as the result of the implementation of different measures. Due to the design of our study and the absence of farm-level information, we could not draw a clear cause-and-effect relationship here, but the association between vaccination and colistin used was evident. Due to unknown reasons, *E. coli* vaccines were underused before 2014 in Estonia, and a simultaneous increase in vaccination and decreased colistin consumption in pigs was confirmed.

The reduction in colistin use could also be explained to some extent by the change in pig herd structure in Estonia over the study period. The number of small pig producers (<3000 pigs) increased 6-hold and the total number of pigs increased by 21% by 2014 compared to 2010. Due to the emergence of African Swine Fever (ASF) in Estonia in 2014 [42], the number of pigs decreased and a large proportion of smaller pig producers ceased production within 2014–2019. Different size pig farms might deviate in regards to their animal health and welfare as well as overall attitudes towards AMU and vaccination. However, by now the number of pigs in Estonia has recovered and remains only about 1% lower than at the start of the current study [43]. In 2015, comprehensive and rigorous biosecurity measures were implemented in pig farms due to ASF and this possibly changed the mentality of the farmers to act more proactively in disease prevention. Due to the personal decision-making process, further studies should be directed to reveal the triggers and motivation of pig farmers and veterinarians to change their farm rearing conditions and treatment practices.

In Estonia, general policymaking decisions about the regulation of AMU at a national level have not been taken. Therefore, all changes in AMU are primarily explained by the behavior and attitude of veterinarians and farmers. The number of veterinarians working in the pig industry is not high in Estonia, and close personal contacts between these groups may support a better understanding of antimicrobial treatment and herd health management, also promoting the use of vaccines. One possible explanation may be the increase of awareness through the educational courses on the topics of prudent use of antimicrobials and improvement of herd health initiated and supported by the Ministry of Rural Affairs. Currently, in Estonia, colistin as a B-category antimicrobial [3] is not indicated for the treatment of animals in the species and disease-specific antimicrobial treatment guidelines.

Many additional factors may affect the reduction of colistin consumption, but which could not be assessed in this study. The increasing need to reduce the use of CIAs in animals necessitates a need to monitor their use based on all available data. The general sales statistics have limited capacity to monitor long-term changes in AMU. There is no available data about the change of consumption of other antimicrobials in those pig farms, where colistin was used. However, the total consumption of antimicrobials in Estonia decreased from 2014 onwards. Further studies should focus on monitoring intervention measures at the farm-level and assessment of associations between AMU and herd health indicators [44]. In addition, we cannot give a clear explanation for the changes in colistin use in cattle and poultry. Due to the relatively small number of farms in Estonia, outbreaks or changes in treatment strategies in single farms can have a pronounced effect on the overall statistics. Due to commercial confidentiality, the specific details were not available for this study. At the national level, the data on the sales of veterinary antimicrobial products are important for guiding and supporting general policymaking decisions. The limitations of our study emphasize the importance of farm-level AMU monitoring, which is critical for driving antimicrobial stewardship, i.e., the establishment and implementation of measures aimed at combatting AMR by promoting responsible AMU practices [45]. In several European countries, farm-level monitoring is already established [46].

Achieving a minimum level of colistin use in food-producing animals avoiding an increase in the use of other antimicrobials and without jeopardizing animal welfare requires the use of preventive measures. Based on summaries of product characteristics (SPC) the primary indication for the oral administration of colistin is the treatment of gastrointestinal infections caused by colistin-sensitive non-invasive *E. coli*. Several studies have shown high AMU in weaned piglets [47,48] and frequent colistin use in piglets and weaners in some countries [49]. Diseases as post-weaning diarrhea could be prevented. Vaccination against *E. coli* could be an important factor implicitly reducing the demand for colistin use in pigs. The European Centre for Disease Prevention and Control (ECDC), the European Food Safety Authority (EFSA), and the EMA joint scientific opinion pointed out that the use of CIAs in food-producing animals is the relevant indicator in assessing the progress in reducing AMU and AMR in humans and animals [50]. Accurate monitoring of diseases for which CIAs are indicated and finding the alternatives might be an effective method to reduce their use.

## 4. Materials and Methods

### 4.1. Collection of Sales Data

Currently available data on the sales of veterinary medicines in Estonia [51] are based on wholesaler’s reports collected by the State Agency of Medicines and include total sales of veterinary medicines to veterinarians and pharmacies. The sales data are collected at package level per each year, including the name of the veterinary medicinal product, identification number of the product (package code), the active ingredient, pharmaceutical form, strength, package size, manufacturer, number of sold packages. Both antimicrobials and vaccination data are presented according to the Anatomical Therapeutic Chemical classification system for veterinary medicines [52].

### 4.2. Collection of Data Allowing Differentiation of Target Species

The national statistics of veterinary medicines of Estonia do not include information on sales by species. The ten years (2010–2019) sales data of colistin-containing products were collected from wholesalers, who sold colistin in those years, as sales to veterinarians treating only pigs, cattle, poultry or other species. Sales to veterinarians working in the mixed veterinary practice (treatment of different animal species) and sales to pharmacies are defined as sales to species unknown (includes sales to cattle, pigs, poultry and other species). Colistin sales data of wholesalers, who could not provide data by species, are defined as sales to species unknown. Due to commercial confidentiality, colistin sales data differentiating cattle and poultry as target species could not be published and the data are therefore merged for these two animal species. Data were collected and calculated according to the same principles as the national statistics.

### 4.3. Calculation of Colistin Consumption Data

Quantities of colistin were calculated for each product by multiplying the number of sold packages by the amount of active ingredient in each package and converted to milligram (mg) active ingredient. The standard conversion factor was used to convert international units (IU) to mg of the active ingredient when strength was given in IU. The number of sold doses of *E. coli* vaccines for pigs was calculated for each product by multiplying the number of sold packages by the number of vaccine doses in each package.

The population correction unit (PCU) was used as a denominator for sales data. The methodology of calculation of PCU is the same as in ESVAC reports [53]. PCU is a technical unit of measurement, which is calculated for each animal category by multiplying numbers of livestock animals and slaughtered animals by the theoretical weight at the time for the treatment.

The PCU (1 PCU = 1 kg of animal biomass) for pigs was calculated as follows:

PCUpigs = PCUdomestic + PCUexport − PCUimport

where, PCUdomestic = number of living sows × estimated weight at treatment + number of slaughtered pigs × estimated weight at the treatment, PCUexport = number of pigs exported for slaughtering or fattening × estimated weight at the treatment and PCUimport = number of pigs imported for slaughtering or fattening × estimated weight at the treatment

For calculation of PCU for pigs, the following standardised average weight in kilograms was used: living sows 240 kg, slaughtered pigs 65 kg, imported-exported pigs for slaughter 65 kg, and imported-exported pigs for fattening 25 kg [53].

For transnational comparison of colistin consumption, the sales data of colistin are expressed in milligrams of active ingredient per kilogram of estimated weight at the treatment of livestock and slaughtered animals (mg/PCU):

$$\mathrm{Total}\;\mathrm{consumption}=\frac{\mathrm{Total}\;\mathrm{amount}\;\mathrm{of}\;\mathrm{sold}\;\mathrm{colistin}\;\mathrm{in}\;\mathrm{mg}}{\mathrm{Total}\;\mathrm{PCU}\;\mathrm{in}\;\mathrm{kg}}$$

The amount of sold colistin for pigs included only sales of colistin to pig farms, and species unknown are not included in the calculations:

$$\mathrm{Consumption}\;\mathrm{in}\;\mathrm{pigs}=\frac{\mathrm{Amount}\;\mathrm{of}\;\mathrm{sold}\;\mathrm{colistin}\;\mathrm{for}\;\mathrm{pigs}\;\mathrm{in}\;\mathrm{mg}}{\mathrm{PCU}\;\mathrm{pigs}\;\mathrm{in}\;\mathrm{kg}}$$

### 4.4. Statistical Analysis

The data of colistin and *E. coli* vaccine consumption over years 2010–2019 was inserted into Microsoft Excel (2016) spreadsheet, and the descriptive analysis was compiled by using the corresponding functions.

In order to analyse the association between colistin and *E. coli* vaccine consumption in pigs in 2010–2019, a time-series analysis was applied by using Stata^®^ MP14.2 (StataCorp, College Station, TX, USA). To sort and index the data by years a ‘tsset’ command was used. A linear model with autoregressive integrated moving average (ARIMA) was created. To estimate the moving-average order (q) a correlogram was composed using the ‘ac’ command. By using a partial correlogram (‘pac’ command) an autoregressive order (p) was specified. The final ARIMA model used colistin consumption (mg/PCU) as an outcome variable, number of *E. coli* doses (×10,000 doses) as a predictor variable, the moving-average order (q) = 0, integrated (difference) order (d) = 1 and autoregressive order (p) = 0.

## 5. Conclusions

The responsible use of antimicrobials promotes avoiding CIAs in food-producing animals and finding alternatives to the highest priority CIAs as colistin. This study revealed a remarkable increase in colistin consumption from 2010 to 2013 and a sharp reduction thereafter in Estonian production animals, whereas that was largely the result of changes in colistin use in pig production. Achieving a minimum level of colistin use in food-producing animals while simultaneously avoiding an increase in the use of other antimicrobials and without jeopardizing animal welfare requires the use of preventive measures. Among other improvements at the farm level, vaccination of piglets against *E. coli* strains could contribute to reduced colistin use in pig production. Accurate monitoring of the diseases for which CIAs are indicated and searching for alternatives might be an effective way to reduce their use.

## Figures and Tables

**Table 1:** Sales of colistin for food-producing animals in Estonia in years 2010–2019.

Sold Colistin	2010	2011	2012	2013	2014	2015	2016	2017	2018	2019
Total sales, kg	378	458	570	709	403	164	95	127	95	53
% of total AM	4.6	5.5	7.3	8.1	4.1	1.7	1.1	1.7	1.3	0.7
Total sales, mg/PCU	3.51	4.31	4.88	5.75	3.12	1.33	0.73	1.14	0.83	n/a
For pigs, kg	336	425	517	651	377	99	86	118	89	42
% of total colistin	89%	93%	91%	92%	93%	60%	91%	93%	93%	79%
For cattle and poultry, kg	22	18	42	32	8	54	4	7	4	6
% of total colistin	6%	4%	7%	5%	2%	33%	4%	5%	5%	12%
For species unknown, kg	20	15	11	26	18	11	5	2	2	5
% of total colistin	5%	3%	2%	4%	5%	7%	6%	2%	2%	9%

**Table 2:** Sales of colistin and *E. coli* vaccines for pigs and number of piglets in Estonia in years 2010–2019.

Characteristic	2010	2011	2012	2013	2014	2015	2016	2017	2018	2019
PCUpigs (in 1000 tonnes)	35	34	43	47	49	45	38	37	40	42
Colistin for pigs (in mg/PCU)	9.63	12.47	12.14	13.88	7.77	2.20	2.27	3.15	2.23	1.00
Produced piglets (in 1000 heads)	752	782	775	750	774	744	592	624	621	n/a
*Escherichia* (in 1000 doses)	0.8	1.7	0.8	2	220	325	209	301	349	362
*Escherichia + Clostridium* (in 1000 doses)	42	75	50	83	83	72	77	67	73	65

## Data Availability

The data presented in this study are available on request from the corresponding author.
